# Dr. Alfred B. Kibbe

**Published:** 1904-05

**Authors:** 


Dr. Alfred B. Kibbe, a graduate of the University of Buffalo, in 1880, died at his home in Seattle, Wash., March 27, 1904.

